# Reconstructing gene-regulatory networks from time series, knock-out data, and prior knowledge

**DOI:** 10.1186/1752-0509-1-11

**Published:** 2007-02-02

**Authors:** Florian Geier, Jens Timmer, Christian Fleck

**Affiliations:** 1 Institute of Physics, University of Freiburg, Hermann-Herder Str. 3, 79104 Freiburg, Germany; 2 Freiburg Center for Data Analysis and Modeling (FDM), University of Freiburg, Eckerstr. 1, 79104 Freiburg, Germany

## Abstract

**Background:**

Cellular processes are controlled by gene-regulatory networks. Several computational methods are currently used to learn the structure of gene-regulatory networks from data. This study focusses on time series gene expression and gene knock-out data in order to identify the underlying network structure. We compare the performance of different network reconstruction methods using synthetic data generated from an ensemble of reference networks. Data requirements as well as optimal experiments for the reconstruction of gene-regulatory networks are investigated. Additionally, the impact of prior knowledge on network reconstruction as well as the effect of unobserved cellular processes is studied.

**Results:**

We identify linear Gaussian dynamic Bayesian networks and variable selection based on F-statistics as suitable methods for the reconstruction of gene-regulatory networks from time series data. Commonly used discrete dynamic Bayesian networks perform inferior and this result can be attributed to the inevitable information loss by discretization of expression data. It is shown that short time series generated under transcription factor knock-out are optimal experiments in order to reveal the structure of gene regulatory networks. Relative to the level of observational noise, we give estimates for the required amount of gene expression data in order to accurately reconstruct gene-regulatory networks. The benefit of using of prior knowledge within a Bayesian learning framework is found to be limited to conditions of small gene expression data size. Unobserved processes, like protein-protein interactions, induce dependencies between gene expression levels similar to direct transcriptional regulation. We show that these dependencies cannot be distinguished from transcription factor mediated gene regulation on the basis of gene expression data alone.

**Conclusion:**

Currently available data size and data quality make the reconstruction of gene networks from gene expression data a challenge. In this study, we identify an optimal type of experiment, requirements on the gene expression data quality and size as well as appropriate reconstruction methods in order to reverse engineer gene regulatory networks from time series data.

## Background

The temporal and spatial coordination of gene expression patterns is the result of a complex integration of regulatory signals at the promotor of target genes [1,2]. In the last years numerous methods have been developed and applied to reconstruct the structure and dynamic rules of gene-regulatory networks from different high-throughput data sources, mainly microarray based gene expression analysis, promotor sequence information, chromatin immunoprecipitation (ChIP) and protein-protein interaction assays [3-6]. Popular reconstruction methods include Bayesian networks [7-9], robust regression [10-12], partial correlations [13-15], mutual information [16,17] and system-theoretic approaches [18,19]. Approaches using gene expression data either focus on static data or on time series of gene expression. The later approach has the advantage of being able to identify causal relations, i.e. gene-regulatory relations, between genes without the need of actively perturbing the system. The reconstruction of gene networks is in general complicated by the high dimensionality of high-throughput data, i.e. many genes are measured in parallel, with only few replicates per gene. Together with observational noise, these complications impose a limit on the reconstruction of gene networks [20,21]. In this study we focus on the following three challenges that a reconstruction of gene-regulatory networks from time series of gene expression data is facing.

• The quality of data derived from high-throughput gene expression experiments is largely limited by noise. For example the typical magnitude of observational noise in microarray measurements is about 20–30% of the signal [22]. In high-throughput techniques dynamical noise maybe expected to play a minor role due to the underlying population sampling of the data. In contrast, data derived from gene expression at the single cell level can exhibit a significant amount of dynamical noise as well as strong cell to cell variations [23].

• Data size, i.e. length of a time series and number of replicates, is limited by the cost of experiments. The typical length of time series measurements in microarray studies is around 10–20 time points [24,25] and 3–5 replicates. Therefore, any model underlying network reconstruction methods must be simple, i.e. contain as few parameters as possible, and robust.

• Gene regulation is due to the activity of transcription factors (TFs) which is in most cases post-translationally controlled by additional factors. This activity is not directly observed by measuring TF expression levels. However, many network reconstruction methods based on time series assume the activity of TFs to be directly related with their expression levels, thereby omitting additional hidden variables [10,26]. Accounting for hidden variables in the framework of network reconstruction methods based on time series demands more data in order to estimate the additional parameters and can complicate a biological interpretation of the hidden variables [27].

A systematic study requires data of several gene regulatory networks where the structure is known in detail. Since no experimental data fulfilling these requirements is currently available we use an ensemble of synthetic gene regulatory networks to generate gene expression data. This approach allows us to investigate in depth the effect of noise, data size and hidden variables in the form of unobserved processes on the reconstruction of gene regulatory networks. We evaluate three methods for the reconstruction of gene regulatory networks from time series which are either based on a discrete or a continuous representation of the network states: discrete dynamic Bayesian networks (discrete DBNs), linear Gaussian DBNs and linear regression in combination with variable selection. All these techniques have been used in former studies, however a comparison of the three methods using temporal gene expression data is missing so far. Time series can be measured under different experimental conditions including changes in the culture conditions or perturbations of network components by gene knock-outs (KOs). For example the time series experiments conducted by Spellman et al. generated data under different culture conditions and using different mutant backgrounds in order to reveal a more comprehensive picture about gene regulation during the yeast cell cycle [24]. We study different experimental design strategies in order to identify optimal experiments for the identification of the underlying network structure. Additionally, we investigate the requirements on data size and data quality that must be met by a successful network reconstruction. Beside gene expression data, other data sources or a combination of them can be called in to reveal the structure of gene-regulatory networks. These data sources include chromatin immuno-precipitation (ChIP) experiments, promoter analysis and protein-protein interaction assays. Within a Bayesian learning framework, these additional data sources can be incorporated as prior knowledge. For example p-values of TF-DNA interactions given by ChIP experiments have been applied as prior knowledge about gene-gene interactions [28]. Here, we investigate the influence of prior knowledge on the reconstruction of gene-regulatory networks. We present a model for prior knowledge based on probabilities for gene-gene interactions. The model is used to generate prior knowledge with different levels of accuracy. The accuracy of the inferred networks is compared to the accuracy of the prior knowledge using different amounts of gene expression data. This allows us to identify conditions when the use of prior knowledge can improve the prediction accuracy.

In a typical study of a gene-regulatory network the states of many molecular components of the network are not observed, such as the phosphorylation-level of proteins or their cellular localization. Using synthetic gene-regulatory networks enables us to study the effect of unobserved processes on the network reconstruction by artificially hiding subsets of the complete data, such as protein levels and promotor states. We investigate the influence of these unobserved states on the identification of the network structure by time series experiments and gene KOs.

The paper is organized as follows. In the first section the generation of the different data sets from an ensemble of 100 synthetic networks is explained. In the second section the reconstruction of the gene networks using linear Gaussian DBNs is studied. Here, we also focus on the optimal type of biological experiment in order to identify the underlying network structure. In section three two alternative network reconstruction methods, discrete DBNs and variable selection based on F-statistics, are evaluated and compared with linear Gaussian DBNs. Section four studies the impact of data sizes and observational noise. In section five we investigate the network reconstruction based on prior knowledge and gene expression data. In the last section the effect of unobserved processes, e.g. protein-protein and protein-DNA interactions, on the structure and identification of gene-gene interaction networks is studied.

## Results and Discussion

### Data generation and evaluation

In order to evaluate the performance of the different network reconstruction methods we generate an ensemble of 100 synthetic networks. This approach allows us to evaluate the average performance of a method without biasing the evaluation in favor of a single network or network feature. Each network consists of 30 genes of which 10 are TFs and 20 are pure target genes. A TF can itself be target gene while a pure target gene is not allowed as a transcriptional regulator of another gene. The distinction between transcriptional regulator and pure target gene allows us to substantially reduce the possible number of regulatory interactions and with it the amount of data needed to identify them. Our approach is also applicable in situations where the total number of genes is much larger compared to the number of involved transcriptional regulators as the computational cost of the network reconstruction methods applied scale linearly with the number of pure target genes (see Methods).

Continuous gene expression data is generated by simulating the network dynamics with non-linear ODEs (see Methods). Observational error is incorporated via an additive-multiplicative error model [29]. This error model corresponds with a first order approximation to log-normal distributed expression levels [30]. In order to identify the optimal data type and experiment for the identification of the underlying network structure we apply different simulation scheme. Either random perturbations from steady state or specific perturbations of the steady state by TF KOs are simulated and time series are sampled during relaxation of the network back to steady state. In order to apply discrete DBNs to the continuous data obtained from ODEs the data is subsequently discretized. Among different discretization scheme we choose binary quantile discretization for which we observe the lowest error rates of the reconstructed networks. For the evaluation of discrete DBNs we also simulate the networks with probabilistic Boolean logic resulting in binary gene expression data. The effect of hidden variables is investigated using a 54-dimensional network of non-linear ODEs which models the interaction of 10 genes with their respective promotor, mRNA and protein states [31]. As above, we generate time series data by single gene KOs and subsequent sampling during relaxation to a new steady state. However, we use only the mRNA data in order to reconstruct the gene-gene interaction network. This approach corresponds to a microarray experiment where the promotor or protein states are not observed.

Based on the studied question, we use two alternative approaches for the evaluation of the reconstructed networks. In the first three sections we evaluate our results by calculating three error rates which are based on an edge-wise comparison of the best reconstructed network with the true network: (1) The false negative rate (FNR) gives the percentage of missed edges from all true edges. (2) The false positive rate (FPR) gives the percentage of predicted edges from all false edges. (3) The realized false discovery rate (FDR) gives the percentage of false edges among the predicted edges (see Methods). In the last two sections we study the effect of prior knowledge and hidden variables. Here, MCMC simulations are applied in order to calculate posterior probabilities for single gene-gene interactions (see Methods). Receiver operating characteristic (ROC) curves and the corresponding area under the ROC curve are used as a measure for the overall accuracy of the network reconstruction based on MCMC simulations.

### Reconstruction of gene networks using linear Gaussian DBNs

**Figure 1:**
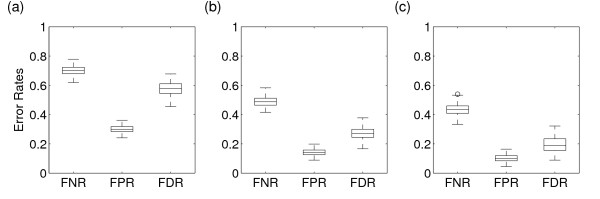
Box plots of error rates of the reconstruction of 100 networks using linear Gaussian DBNs. Each network consists of 30 genes. Error rates are determined by comparing the best reconstructed network with the true network. All three plots are based on the same number of data points per gene. The boxes show lines at the lower quartile, median, and upper quartile values. Outliers are indicated by open circles. (a) One time series replicate with 40 data points per gene based on a random perturbation of the steady state. (b) 10 independent time series replicates each with 4 data point per gene based on random perturbations of the steady state. (c) 10 time series replicates of length 4 each based on single TF knock-outs. FNR: false-negative rate. FPR: false-positive rate. FDR: realized false discovery rate. The observational noise level is 2%.

We start our investigation by evaluating the reconstruction of 100 gene regulatory networks using linear Gaussian DBNs. Figure 1(a) shows box plots of the three error rates of network reconstruction based on data of a single time series for each network. Each time series is generated by a random perturbation of the corresponding network from its steady state and consists of 40 data points per gene. The observational noise level is 2%. As indicated by the high error rates, the average network reconstruction from this data sets is very poor. Almost 60% of the predicted edges are wrong (see FDR, Figure 1(a)), while only 30% of the true interactions are also predicted (see FNR, Figure 1(a)). Most of the network structures cannot be identified although the temporal resolution of the underlying data is relatively high due to a high sampling rate. In Figure 1(b) we change the sampling rate and the number of replicates. Here, for each network we generate 10 time series with 4 data points each as opposed to the previous approach where one time series with 40 data points per network is used. Thus, the total size of the data is equivalent in both approaches. However, for each of the 10 time series the steady states of the networks are perturbed independently. The resulting error rates are considerably smaller. Only about 30% of the predicted edges are wrong while about 50% of all true edges are also discovered. These results clearly indicate that several random perturbations contain more information about the gene regulatory interactions compared to a single time series with an equivalent data size, i.e. with a higher sampling rate. In general perturbations are necessary to push the gene regulatory network out of its steady state. However, a single perturbation of all genes is unlikely to reveal the regulatory impact of all TFs onto their target genes as some of these TFs will be perturbed in a similar manner. Therefore several uncorrelated perturbations are necessary to distinguish between the regulatory impact of each TF onto its target genes.

However, conducting several independent, random perturbation of all the components of a gene-regulatory network is not experimentally feasible. Moreover, perturbations which change the structure and function of the underlying network unspecifically should be avoided. We therefore study specific perturbations of the gene regulatory networks in the form of TF knock-outs (KOs). Each of the 10 TFs in the 100 synthetic networks is knocked out separately and time series are sampled while the system relaxes back to its new steady state. The resulting error rates of the network reconstructions are shown in Figure 1(c). All three error rates are significantly smaller (t-test on all three rates, *p *< 10^-16^) compared to the error rates based on random perturbations as shown in Figure 1(b). On average only 20% of the predicted edges are wrong while almost 60% of all true interactions are discovered. Thus, specific TF KOs additionally improve the identification of the network structures. The additional gain over random perturbations as in Figure 1(b) can be explained by the specificity of the TF KOs. The perturbations applied in Figure 1(b) change the levels of all genes at the same time and do not reveal the specific impact of a single TF. In contrast, our results indicate that a perturbation by TF KOs gives the required specificity in order to reveal the regulatory connections between the genes. Depending on the biological system studied, several TF KOs represent a considerable experimental effort in order to achieve independent and specific perturbations of the biological system. High-throughput RNAi based gene KOs are an attractive possibility to generate the data required for a successful reconstruction of gene-regulatory networks [32].

In a study based on static Bayesian networks Werhli et al. [33] come to a similar the conclusion. They show that active interventions improve the identification of the network structure over an inference solely based on passive observations. Here, active interventions are necessary in order to resolve the ambiguity of certain edge directions intrinsic to the inference of network structures based on static and passive observations. Active interventions can break the symmetry of correlations between nodes in a network and identify the causal, i.e. assymmetric, relation between the nodes. It is important to note that the improvement due to TF KOs we observe is not due to a similar phenomenon as observed by Werhli et al. for static Bayesian networks. All edges in the DBNs we apply are directed in time and represent causal relations *per se*. The gain of TF KOs over a single replicate time series as in Figure 1(a) is only due to the larger number of independent replicates in the form of specific perturbations of the underlying network. This can be already seen by the improvements of the error rates from Figure 1(a) to 1(b).

### Reconstruction of gene networks with alternative methods: discrete DBNs and variable selection

**Figure 2:**
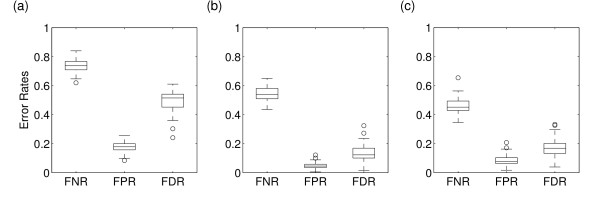
Box plots of error rates of the reconstruction of 100 networks using discrete DBNs (a-b) or variable selection based on F-statistics (c). The network reconstruction is based on: (a) Binarily discretized data underlying Figure 1(c). (b) Binary data generated by simulating the networks with probabilistic Boolean logic. 10 time series with 4 data points per gene are used. (c) Same data underlying Figure 1(c) but using variable selection for the network reconstruction.

In this section we compare linear Gaussian DBNs to more commonly used and related methods to reconstruct gene regulatory networks from time series. First, we focus on discrete DBNs. The data underlying Figure 1(c) is discretized prior to the application of discrete DBNs. We use a binary quantile discretization. The resulting error rates are shown in Figure 2(a). It turns out that the overall performance of discrete DBNs is rather poor compared with the performance of linear Gaussian DBNs on the corresponding continuous data. The average FDR is 50% meaning that half of the predicted edges are wrong. Only about 25% of all true gene-gene interactions are also identified. Both error rates are considerably higher than the corresponding error rates of linear Gaussian DBNs (compare Figure 1(c) and Figure 2(a)). These results suggest that discretization of the continuous data leads to a large information loss. In order to improve our results we also tested a different discretization method, i.e. ternary quantile discretization in combination with an information preserving coalescence of discretization levels [34]. We observe no significant improvement between the corresponding FDRs (data not shown). Data discretization leads to a qualitative and coarse representation of the dynamics. Different time scales of the dynamics cannot be reflected without considering a finer sampling rate and a higher number of discretization levels. However, more discretization levels also require an increase in data size in order to identify all the necessary parameters of the corresponding multinomial conditional probability distributions. For the data sizes and networks studied, we observe a considerably better performance of linear Gaussian DBNs compared to discrete DBNs despite the fact that linear Gaussian DBNs make stronger assumptions about the underlying dynamics. We therefore conclude that the advantage of discrete DBNs to capture also non-linear effects can only be utilized with much larger data sizes than we consider and that are usually available in a time series microarray experiment.

Alternative to the discretization of continuous data is the simulation of the network dynamics by probabilistic Boolean logic. We simulate the dynamics by applying simple activation and inhibition rules to the regulation of a gene and combine the regulation by different TFs in a logic OR gate. We also incorporate dynamical noise into the simulation in form of random switches in the outcome of the regulatory rules (see Methods). In Figure 2(b) we show the corresponding error rates based on the network reconstruction from Boolean data. As one can see, the overall performance of discrete DBNs on Boolean data has much improved. Especially the average FDR is very low, i.e. about 10%. The FNR indicates that 55% of all true edges are identified on average. These results are considerably better than the performance of discrete DBNs on the discretized data in Figure 2(a) and the FPR and FDR are significantly better as the corresponding rates of linear Gaussian DBNs on the continuous data in Figure 1(c) (T-test: FPR, *p *< 10^-16^; FDR, *p *= 10^-13^). These results might question which of the two modeling frameworks, continuous ODEs or Boolean logic in combination with dynamical noise, accurately reflect cellular dynamics? Boolean networks are a very crude representation of the time-continuous cellular dynamics underlying gene regulatory networks. Especially the different time scales involved in the regulation of genes cannot be described by Boolean networks without considering additional hidden variables. In contrast, non-linear ODEs are a natural framework for the description of cellular processes as they incorporate time scales as well as the concentration of transcripts directly. The consideration of dynamical noise is important in order to understand the dynamics on a single cell level [23]. Here, dynamical noise can play a role due to the low concentrations of molecular species and random thermal fluctuations of the cellular environment. Additionally, cell to cell variations in the kinetic parameters, e.g. induced by variable expression levels of the enzymes, are important. However, high-throughput techniques based on population sampling cannot reveal a single cell resolution in a time series. Here, observational noise plays the dominant role. Based on our results we suggest the use of linear Gaussian DBNs as they better fit the data generated by high-throughput experiments.

Next we investigated an alternative network reconstruction method that can be applied to continuous time series data: variable selection based on F-statistics. The method we use is based on a linear regression of the TF expression levels at time *t *against the expression levels of each target gene at time *t *+ 1. In an repetitive step-wise selection and elimination procedure a set of optimal predictors, i.e. TFs, is build for each target gene according to a partial F-test. The TF with the significantly highest partial correlation coefficient is included in the set of predictors, while the TF in the set with the lowest, non-significant partial correlation coefficient is excluded. This step-wise selection procedure leads to a local optimization of the set of TFs for each gene. Figure 2(c) shows the corresponding error rates of the networks reconstructed by variable selection based on the data underlying Figure 1(c). All three error rates are on average similar to the error rates of the reconstruction using linear Gaussian DBNs. The average FNR is 50%, thus about half of the true gene-gene interactions are identified. At the same time about 15% false interactions are predicted. The overall good performance of the variable selection procedure seen in Figure 2(c) can be attributed to the relatively large data size (= 40 data points per gene) and the limited number of regressors (10 TFs) as well as the simplicity of the regulatory model which excludes any interactions between TFs. It is important to note, that the realized error rates are governed by the parameters of the F-statistics and the significance level *α *of the partial correlations between the expression levels of TFs and target genes in the final network. In the given example *α *≤ 0.01 is chosen to give a FNR comparable with Figure 2(b). Larger significance levels lead to a larger FPR and FDR. Thus, in order to get low error rates only TFs with a small p-value are included in the network. One has to keep in mind that a clear interpretation of the p-values is not possible due to the problem of multiple testing.

### The effect of data size and noise

**Figure 3:**
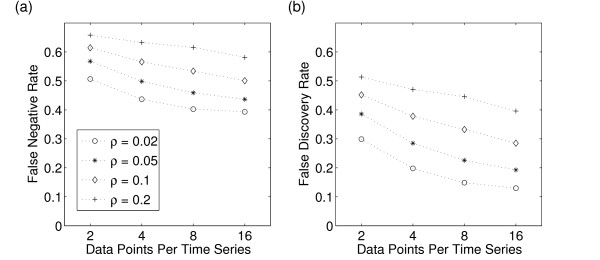
The relation between data size and observational noise. Average false-negative (FNR) and realized false discovery rates (FDR) for different levels of observational noise in dependence of the data size. Observational noise follows an additive-multiplicative Gaussian error model with a common standard deviation *ρ*. The results are based on the reconstruction of 100 networks. The data is generated by KOs of all 10 TFs in each network and sampling a different number of time points per KO. For example two data points per time series correspond with 20 data points per gene in total.

In this section we investigate the impact of data size and observational noise on the reconstruction of gene regulatory networks by linear Gaussian DBNs. Figure 3 shows the average FNRs and FDRs of the reconstruction of 100 networks with respect to data size and different observational noise levels. As in Figure 1(c), the underlying data is generated by single KO experiments of all 10 TFs of a given network. The data size is varied by using different sampling rates in the TF KO experiments. On the x-axis of Figure 3(a–b) the length of the time series of a single TF KO experiment is given. A time series of length 2 indicates a total data size of 20 time points per gene, i.e. 2 data points per time series × 10 TF KO experiments. As expected, the error rates increase with the noise level but decrease with data size.

Conducting 10 TF KO experiments each with 2 data points per gene leads to a FDR of about 50% given a measurement error in the order of 20%. The FDR can be lowered in two ways. A FDR of 40% is achieved either by halving the measurement error to 10% or by measuring longer time series, i.e. 16 data points per gene. As the measurement error is intrinsic to the experimental method, our results can be interpreted as an estimate for the required number of data points per gene needed in order to identify a network structure of a certain quality. It is important to note that the TF KO experiments underlying Figure 3 represent an optimal experimental design for the identification of the true network structure. As mentioned above, a single time series of an equivalent data size contain much less information about the underlying network structure. Therefore our estimates represent only a lower bond for the required number of data points per gene if other design strategies are used.

Realistic observational noise levels of microarray experiments are in the order of 20%–30% of the signal [22] while the data size of time series experiments usually range from 10–20 time points per gene [24,25]. Our results indicate, that network reconstruction with currently available data will still give rise to many false predictions (FDR ~ 50%).

### The influence of prior knowledge

Prior knowledge can potentially improve the accuracy of the inferred networks [28]. However, the degree of improvement depends on the quality of the prior knowledge as well as the amount of available gene expression data. In this section we investigate the benefits of the use of prior knowledge in form of prior probabilities for gene-gene interactions within a Bayesian learning scheme. We compare the accuracy of a network reconstruction based on prior knowledge alone with the accuracy of a reconstruction based on the corresponding posterior probabilities calculated by MCMC simulations. This enables us to predict under what circumstances, i.e. quality of the prior, amount of gene expression data, the use of prior knowledge can be a benefit for the reconstruction of gene regulatory networks.

**Figure 4:**
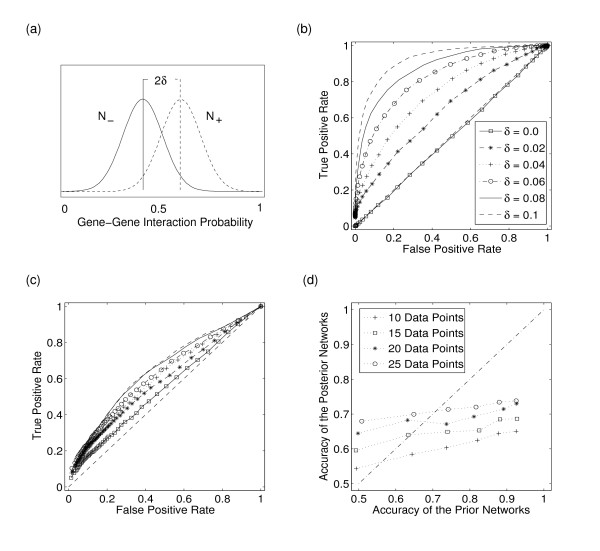
The benefit of using prior knowledge. Reconstruction of 20 networks based on MCMC simulations using prior knowledge and gene expression data. The reconstruction is evaluated using receiver operating characteristic (ROC) curves and the area under the ROC curve (AUC) as a measure for the accuracy of the reconstruction. (a) The prior knowledge model used to generate prior gene-gene interaction probabilities for true (*N*_+_) and false edges (*N*_-_) respectively. The parameter *δ *controls the accuracy of the prior knowledge by determining the separation of the means of both truncated Gaussian probability distributions. (b) Average ROC curves of a network reconstruction based on prior gene-gene interaction probabilities alone. Each curve represents a different level of *δ*. The dashed curve indicates the performance of a random prediction. It corresponds with an accuracy (AUC) of 0.5. (c) Average ROC curves of a network reconstruction based on prior knowledge and gene expression data. The size of the expression data is 10 data points per gene. The posterior probabilities are calculated by MCMC simulations. Each curve represents a different level of accuracy of the prior knowledge and corresponds with the prior knowledge used in panel (b). (d) Average accuracy of the prior networks versus average accuracy of the posterior networks for different data sizes. The dashed-dotted line indicates the equivalence between prior and posterior accuracy.

We develop a simple model where the prior knowledge is given by the probability of a gene-gene interaction based on two probability distributions for true and false interactions respectively. The model is depicted in Figure 4(a). The prior interaction probabilities are drawn from two truncated normal distributions, *x *_±_ ~ *N *_±_ (*μ *± *δ*, *σ*) where *N*_+ _is the probability distribution for true interactions and *N*_- _is the probability distribution for false interactions. The parameters *μ *is set to 0.5 and the standard deviation *σ *is set to 0.1. The accuracy of the prior knowledge is controlled by the parameter *δ *which is used to separate the means of both distributions. We use the model to generate prior probabilities for a subset of 20 networks each consisting of 30 genes. The accuracy of the prior knowledge is calculated using receiver operating characteristic (ROC) curves. ROC curves display the TPR in dependence of the FPR. The TPR and FPR are determined in dependence of a prediction threshold for the prior probabilities; edges with a probability above the threshold are included in the network. The accuracy corresponds with the area under the ROC curve (AUC). A random prediction has an accuracy of 0.5 meaning that on average the prediction cannot distinguish between true and false edges.

Figure 4(b) shows average ROC curves based on the prior probabilities for different levels of *δ*. For *δ *= 0 the average ROC curve corresponds with the ROC curve of a random prediction (dashed curve in Figure 4(b)). This is expected as the distribution of the prior probabilities for true and false edges are the same for *δ *= 0. With increasing *δ *the average predicting accuracy increases. For *δ *= 0.1, a prediction threshold corresponding to a TPR of 80% leads to 10% false-positives. Thus, a prediction based on this prior knowledge alone leads already to very accurate networks.

Figure 4(c) shows average ROC curves based on the posterior probabilities of gene-gene interactions. These posterior probabilities are determined by MCMC simulations using gene expression data and prior knowledge (see Methods). The data size of the gene expression data is 10 data points per gene. If the prior knowledge on gene-gene interactions is uninformative, i.e. *δ *= 0, the mean accuracy (i.e. mean AUC) is about 55%. Thus, on average 10 data points can improve the random prediction of the uninformative prior. Increasing the accuracy of the prior, by increasing the parameter *δ*, also increases the accuracy of the posterior networks. With *δ *= 0.1 and a data size of 10 data points per gene the average posterior accuracy is 65%. This shows that the use of prior knowledge can improve the prediction accuracy compared to a prediction based on gene expression data alone.

It is interesting to compare whether the posterior networks are always of a higher accuracy compared to the accuracy of the prior networks. Figure 4(d) depicts the average accuracy of the prior networks versus the average accuracy of the posterior networks for different data sizes. Points above the dashed line show an increase in accuracy of the posterior networks relative to the accuracy of the prior networks. Points below the dashed line indicate a respective decrease in accuracy. The four curves in Figure 4(d) depict the dependency between prior and posterior accuracy for four different data sizes. The bisecting line indicates the situation when no gene expression data is used: prior and posterior accuracy are the same. If gene expression data is included the slope of the curves decreases from one. This can be explained by the fact that the posterior probability of a network structure is given by the product of the prior and the likelihood (see Equation 5). As the prior is data-independent and the likelihood is proportional to the data size, the impact of the prior onto the posterior decreases with data size. This implies, that the slope of the curves decrease with larger data sizes. This can be seen in Figure 4(d) by comparing the slopes of the curve corresponding to 10 and 25 data points. The slope of the curve will approach one in the limit of small gene expression data as in this case the likelihood of the data will also approach one.

The average accuracy of the networks predicted by gene expression data alone, i.e. by using an uninformative prior, corresponds with the leftmost points in the graph. For example the average accuracy of networks predicted by using 25 data points per gene is 68%. This prediction can be increased by using more informative priors. However, this increase in accuracy is relatively weak as indicated by the slope of the curves. Once the curves cross the dashed line, prior knowledge alone gives a better prediction than a combination of prior knowledge and gene expression data. This indicates that the use of a combination of prior knowledge and gene expression data within a Bayesian learning framework is not always of an advantage. Only situations where the amount of gene expression data is very limited can show a considerable increase in accuracy by the use of prior knowledge. However, under these data situations a prediction based on the prior knowledge alone might lead to more accurate networks. As the prior and posterior accuracy cannot be determined using real data, it is not possible to decide whether a combination of prior knowledge and gene expression data will give any benefit. Our results indicate that the overall gain by combining data in a Bayesian learning framework, i.e. data situations corresponding to points above the dashed line in Figure 4(d), is limited.

Nevertheless, prior knowledge can be of a benefit in the reconstruction of networks from time series. It can be used to restrict the number of possible gene-gene interactions, e.g. by allowing gene-gene interactions only between TFs and target genes which have a significant prior probability. This improves heuristic search procedures as it restricts the space of the possible posterior models (see Methods). However, if detailed information on the promotor structure is available, alternative approaches which do not use the temporal information of the gene expression data explicitly are even more appropriate [35-37].

### The effect of hidden variables

**Figure 5:**
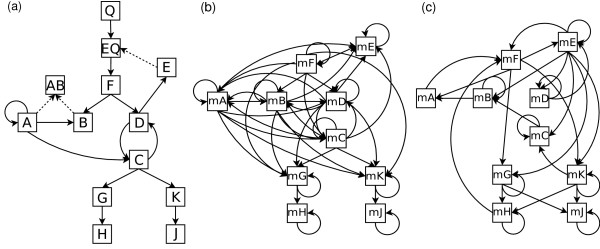
The influence of hidden variables. (a) Transcriptional-regulatory network as given in [31]. Nodes correspond to genes. Solid lines indicate transcriptional regulation, dashed lines represent interactions between proteins. (b) Connectivity of the observed mRNA variables. The network is constructed from the Jacobian matrix of the system of ODEs given by [31]. (c) mRNA interaction network reconstructed from KO time series of all 10 genes; each time series contributes 10 data points per gene. MCMC simulations are used to calculate mRNA-mRNA interaction probabilities. Only interactions with a posterior probability ≥ 0.5 are depicted.

A gene-regulatory network is always integrated into a larger biochemical network which also includes proteins, small signalling molecules and regulated transport between cellular compartments. These processes and quantities are usually not resolved or measured parallel to expression studies, i.e. they are hidden to the experimentator. We study the effect of hidden variables using a gene-regulatory network developed by [31] which is based on a coupled system of ODEs for 54 variables including mRNAs, proteins and promotors. Figure 5(a) shows the structure of the gene-gene interaction network as given in [31]. Nodes in the graph represent genes, solid edges represent interactions based on direct transcriptional regulation, i.e. the mRNA product of the gene is a TF which binds to the promoter of the respective target gene and regulates its expression whereas the dotted edges represent protein-protein interactions. All the depicted interactions are indirect in the system of ODEs, since the interaction between genes is communicated by their products and promotor states. Thus, Figure 5(a) is an abstraction of the underlying physical model. Since our network reconstruction method assumes direct dependencies via the first order Markov assumption of fully-observed DBNs, we cannot expect that the recovered structure resembles the network in Figure 5(a). But we can deduce a more realistic reference network from the system of ODEs. All direct interactions in the system of ODEs are defined by the structure of corresponding Jacobian matrix. This structure can be used to deduce an interaction graph for the subset of observed variables, i.e. mRNAs (see Methods). Figure 5(b) displays the structure of the model reduced to the observed mRNA components. Again, all shown interactions in the network are indirect. Interpreting mRNA nodes as gene nodes, all interactions present in Figure 5(a) are also present in Figure 5(b). However, many more indirect interactions arise. All of them have a clear physical justification: a change in the level of one mRNA *directly *influences the levels of the corresponding target mRNAs. Therefore, the edges in the network of Figure 5(b) correspond either to direct transcriptional regulation (solid edges in Figure 5(a)) or indirect regulation due to unobserved processes. Because of the first order Markov assumption underlying our method we expect, that all of the edges in Figure 5(b) can be recovered by a network reconstruction based on time series data.

Figure 5(c) shows a network recovered from time series data generated under single KOs of all 10 genes using linear Gaussian DBNs and MCMC simulations in order to calculate posterior probabilities for the mRNA-mRNA interactions (see Methods). All depicted edges have a posterior probability ≥ 0.5. We choose this threshold since all remaining edges have a significantly lower probability. Only 3 out of 28 predicted edges are based on TF-mediated gene regulation and are also present in Figure 5(a). However, these edges are not among the most likely edges. In contrast, 15 out of 28 predicted edges are also in the network of Figure 5(b). Most of these edges represent self regulation. Some of the edges are due to indirect protein-protein or protein-DNA interactions. E.g. the regulation *B *→ *A *is reconstructed with high probability, *P*(*B *→ *A*) = 0.89. It is due to the unobserved protein interaction of *A *and *B *(see dotted lines in Figure 5(a)). *A *KO of gene *B *strongly affects the steady state level of gene *A *by lowering the overall degradation of the positive regulator *A*. Thus *B *is a negative regulator of gene *A*. The recovered edge reflects an important regulation within the network, which would have been missed, if gene *B *was classified as a pure target gene based on prior knowledge. For example many methods that combine ChIP data with gene expression data can only recover interactions between direct transcriptional regulators and their target genes [35-37]. In order to identify these unobserved processes, we suggest to use prior knowledge, such as gene ontologies, to identify the set of possible transcriptional regulators. Regulators predicted from time-series which are not classified as transcriptional regulators might indicate unobserved regulatory processes.

Our results indicate that networks reconstructed from time series gene expression data can contain many regulatory interactions which are not based on direct transcriptional regulation, but reflect regulation due to unobserved processes, e.g. protein-protein interactions. A biological example of a process, unobserved in microarray experiments that is crucial for the regulation of target genes involves the *SWI4*/*SWI6 *(SBF) controlled transcription of G1-specific genes during the yeast cell cycle. Phosphorylation of the SBF repressor WHI5 by CLN3/CDK1 leads to a derepression of G1-specific transcription [38]. The transcriptional activity of the SBF complex is not determined by the expression level of its components. In terms of reconstructing gene networks this means that the first order Markov assumption of DBNs, i.e. a relation between the expression level of a transcription factor and its regulatory potential, is not fulfilled due to hidden variables. Alternative approaches include hidden variables directly into their model in order to increase the prediction accuracy [27]. However, a clear biological interpretation of these additional variables is difficult, as the nature and number of the hidden variables is usually unknown.

The hidden activity of TFs can be revealed by relating TF binding sites in promotor regions of target genes with the expression levels of the target genes [35-37]. The crucial difference between these approaches and the approach studied in this paper is that the former group genes with similar TF binding sites and use their expression levels as measurement replicates for the reconstruction of the hidden TF activity. These approaches have less requirements on the number of data points per gene, but are only applicable if additional information about the promotor structure of genes is available. Also, temporal information is not explicitly used.

In order to reconstruct directed gene regulatory networks, i.e. networks that distinguish between regulator and target, from gene-expression data alone, either time series experiments, several gene KO experiments or, as indicated by this study, a combination of both must be conducted. However, our results indicate an inherent limitation to the reconstruction of gene-gene networks by gene expression data alone as the recovered edges between genes do not necessarily reflect direct transcriptional regulations.

## Conclusion

In this study we identify suitable reconstruction methods, types of experiments and requirements on data size and quality in order to reverse engineer gene regulatory networks from time series data. Our results suggest that linear Gaussian DBNs and variable selection are both appropriate methods to reconstruct the network structure from time series of gene expression data. Discrete DBNs are well suited for the reconstruction of probabilistic Boolean networks. However, we find that their ability to reconstruct networks from discretized gene expression data is limited by their higher requirements on data size. In order to optimally identify the structure of gene-regulatory networks we show that experimental data should be generated while specifically perturbing the underlying network. We suggest TF KOs as specific perturbations that allow a network reconstruction from relatively short time series.

The trade-off between observational noise and data size is described and estimates for the amount of data needed in order to reconstruct accurate networks given a certain level of observational noise are provided. For example at least 20 data points per gene are necessary in order to perform better than a random prediction given observational noise levels of 20% which corresponds with the noise level of data commonly derived from microarray measurements.

We identify conditions under which prior knowledge can improve the prediction accuracy. The benefit of prior knowledge within a Bayesian learning framework is limited to a particular data setting where only a small amount of gene expression data is available.

We show that unobserved cellular processes lead to the reconstruction of regulatory relations between genes which are not based on direct transcriptional regulation. The ambiguity of the regulatory relations represent an inherent limitation to the reconstruction of gene-regulatory networks from time series of gene expression.

## Methods

### Network reconstruction

Dynamic Bayesian networks (DBNs) are used to model the stochastic evolution of a set of random variables. The assumption underlying DBNs is a first-order Markov dependence: the state of each variable depends only on the state of its immediate effectors, i.e. it's parents at the previous time point. This assumption can be formalized in the following factorization of the joint probability distribution of a DBN:

P(x=D)=P(x1=D1)∏t=1T−1∏i=1NP(xit+1=Dit+1|pa[xi]t=Dpa[xi]t).     (1)
 MathType@MTEF@5@5@+=feaafiart1ev1aaatCvAUfKttLearuWrP9MDH5MBPbIqV92AaeXatLxBI9gBaebbnrfifHhDYfgasaacH8akY=wiFfYdH8Gipec8Eeeu0xXdbba9frFj0=OqFfea0dXdd9vqai=hGuQ8kuc9pgc9s8qqaq=dirpe0xb9q8qiLsFr0=vr0=vr0dc8meaabaqaciaacaGaaeqabaqabeGadaaakeaacqWGqbaucqGGOaakieqacqWF4baEcqGH9aqpcqWFebarcqGGPaqkcqGH9aqpcqWGqbaucqGGOaakcqWF4baEdaahaaWcbeqaaiabigdaXaaakiabg2da9iab=reaenaaCaaaleqabaGaeGymaedaaOGaeiykaKYaaebCaeaadaqeWbqaaiabdcfaqjabcIcaOiabdIha4naaDaaaleaacqWGPbqAaeaacqWG0baDcqGHRaWkcqaIXaqmaaGccqGH9aqpcqWGebardaqhaaWcbaGaemyAaKgabaGaemiDaqNaey4kaSIaeGymaedaaOGaeiiFaWNaemiCaaNaemyyaeMaei4waSLaemiEaG3aaSbaaSqaaiabdMgaPbqabaGccqGGDbqxdaahaaWcbeqaaiabdsha0baaaeaacqWGPbqAcqGH9aqpcqaIXaqmaeaacqWGobGta0Gaey4dIunaaSqaaiabdsha0jabg2da9iabigdaXaqaaiabdsfaujabgkHiTiabigdaXaqdcqGHpis1aOGaeyypa0Jaemiraq0aa0baaSqaaiabdchaWjabdggaHjabcUfaBjabdIha4naaBaaameaacqWGPbqAaeqaaSGaeiyxa0fabaGaemiDaqhaaOGaeiykaKIaeiOla4IaaCzcaiaaxMaadaqadaqaaiabigdaXaGaayjkaiaawMcaaaaa@782D@

Here, **x **denotes a vector of *N *random variables measured at *T *time points; xit
 MathType@MTEF@5@5@+=feaafiart1ev1aaatCvAUfKttLearuWrP9MDH5MBPbIqV92AaeXatLxBI9gBaebbnrfifHhDYfgasaacH8akY=wiFfYdH8Gipec8Eeeu0xXdbba9frFj0=OqFfea0dXdd9vqai=hGuQ8kuc9pgc9s8qqaq=dirpe0xb9q8qiLsFr0=vr0=vr0dc8meaabaqaciaacaGaaeqabaqabeGadaaakeaacqWG4baEdaqhaaWcbaGaemyAaKgabaGaemiDaqhaaaaa@311E@ is the i-th variable at time *t*. **D **is the data matrix (i.e. time series of gene expression values). *P*(**x **= **D**) is the joint probability of **x **being in state **D**. P(xit+1=Dit+1|pa[xi]t=Dpa[xi]t)
 MathType@MTEF@5@5@+=feaafiart1ev1aaatCvAUfKttLearuWrP9MDH5MBPbIqV92AaeXatLxBI9gBaebbnrfifHhDYfgasaacH8akY=wiFfYdH8Gipec8Eeeu0xXdbba9frFj0=OqFfea0dXdd9vqai=hGuQ8kuc9pgc9s8qqaq=dirpe0xb9q8qiLsFr0=vr0=vr0dc8meaabaqaciaacaGaaeqabaqabeGadaaakeaacqWGqbaucqGGOaakcqWG4baEdaqhaaWcbaGaemyAaKgabaGaemiDaqNaey4kaSIaeGymaedaaOGaeyypa0Jaemiraq0aa0baaSqaaiabdMgaPbqaaiabdsha0jabgUcaRiabigdaXaaakiabcYha8jabdchaWjabdggaHjabcUfaBjabdIha4naaBaaaleaacqWGPbqAaeqaaOGaeiyxa01aaWbaaSqabeaacqWG0baDaaGccqGH9aqpcqWGebardaqhaaWcbaGaemiCaaNaemyyaeMaei4waSLaemiEaG3aaSbaaWqaaiabdMgaPbqabaWccqGGDbqxaeaacqWG0baDaaGccqGGPaqkaaa@5426@ is a conditional probability distribution describing the dependence of the state of the i-th component of **x **at time *t *+ 1 on the state of its parents at time *t*. Equation 1 defines a first-order auto-regressive process in time. Any instantaneous or higher order dependencies are excluded. The factorization of Equation 1 corresponds with a graphical representation where variables are represented as nodes and edges between variables are defined by the conditional probabilities. Learning the structure of a DBN is equivalent to finding a factorization of Equation 1 which maximizes a certain network score given some data instance. The Bayesian scoring metric as introduced by [39] is used to evaluate the structure of a DBN. It is given by log*P*(ℳ
 MathType@MTEF@5@5@+=feaafiart1ev1aaatCvAUfKttLearuWrP9MDH5MBPbIqV92AaeXatLxBI9gBamrtHrhAL1wy0L2yHvtyaeHbnfgDOvwBHrxAJfwnaebbnrfifHhDYfgasaacH8akY=wiFfYdH8Gipec8Eeeu0xXdbba9frFj0=OqFfea0dXdd9vqai=hGuQ8kuc9pgc9s8qqaq=dirpe0xb9q8qiLsFr0=vr0=vr0dc8meaabaqaciaacaGaaeqabaWaaeGaeaaakeaaimaacqWFZestaaa@3790@|**D**) = log*P*(**D**|ℳ
 MathType@MTEF@5@5@+=feaafiart1ev1aaatCvAUfKttLearuWrP9MDH5MBPbIqV92AaeXatLxBI9gBamrtHrhAL1wy0L2yHvtyaeHbnfgDOvwBHrxAJfwnaebbnrfifHhDYfgasaacH8akY=wiFfYdH8Gipec8Eeeu0xXdbba9frFj0=OqFfea0dXdd9vqai=hGuQ8kuc9pgc9s8qqaq=dirpe0xb9q8qiLsFr0=vr0=vr0dc8meaabaqaciaacaGaaeqabaWaaeGaeaaakeaaimaacqWFZestaaa@3790@) + log*P*(ℳ
 MathType@MTEF@5@5@+=feaafiart1ev1aaatCvAUfKttLearuWrP9MDH5MBPbIqV92AaeXatLxBI9gBamrtHrhAL1wy0L2yHvtyaeHbnfgDOvwBHrxAJfwnaebbnrfifHhDYfgasaacH8akY=wiFfYdH8Gipec8Eeeu0xXdbba9frFj0=OqFfea0dXdd9vqai=hGuQ8kuc9pgc9s8qqaq=dirpe0xb9q8qiLsFr0=vr0=vr0dc8meaabaqaciaacaGaaeqabaWaaeGaeaaakeaaimaacqWFZestaaa@3790@) + const. where ℳ
 MathType@MTEF@5@5@+=feaafiart1ev1aaatCvAUfKttLearuWrP9MDH5MBPbIqV92AaeXatLxBI9gBamrtHrhAL1wy0L2yHvtyaeHbnfgDOvwBHrxAJfwnaebbnrfifHhDYfgasaacH8akY=wiFfYdH8Gipec8Eeeu0xXdbba9frFj0=OqFfea0dXdd9vqai=hGuQ8kuc9pgc9s8qqaq=dirpe0xb9q8qiLsFr0=vr0=vr0dc8meaabaqaciaacaGaaeqabaWaaeGaeaaakeaaimaacqWFZestaaa@3790@ defines the factorization of Equation 1 and thus corresponds to the graphical structure of the DBN. *P*(ℳ
 MathType@MTEF@5@5@+=feaafiart1ev1aaatCvAUfKttLearuWrP9MDH5MBPbIqV92AaeXatLxBI9gBamrtHrhAL1wy0L2yHvtyaeHbnfgDOvwBHrxAJfwnaebbnrfifHhDYfgasaacH8akY=wiFfYdH8Gipec8Eeeu0xXdbba9frFj0=OqFfea0dXdd9vqai=hGuQ8kuc9pgc9s8qqaq=dirpe0xb9q8qiLsFr0=vr0=vr0dc8meaabaqaciaacaGaaeqabaWaaeGaeaaakeaaimaacqWFZestaaa@3790@) is the prior probability of the network structure and *P*(**D**|ℳ
 MathType@MTEF@5@5@+=feaafiart1ev1aaatCvAUfKttLearuWrP9MDH5MBPbIqV92AaeXatLxBI9gBamrtHrhAL1wy0L2yHvtyaeHbnfgDOvwBHrxAJfwnaebbnrfifHhDYfgasaacH8akY=wiFfYdH8Gipec8Eeeu0xXdbba9frFj0=OqFfea0dXdd9vqai=hGuQ8kuc9pgc9s8qqaq=dirpe0xb9q8qiLsFr0=vr0=vr0dc8meaabaqaciaacaGaaeqabaWaaeGaeaaakeaaimaacqWFZestaaa@3790@) is its marginal likelihood. An important feature of the Bayesian score is its decomposability: the score of a DBN is the sum of the scores of the log conditional probabilities for each node. The calculation of the Bayesian score for a given DBN and data instance is based on the marginal likelihood of the data,

*P*(**D**|ℳ
 MathType@MTEF@5@5@+=feaafiart1ev1aaatCvAUfKttLearuWrP9MDH5MBPbIqV92AaeXatLxBI9gBamrtHrhAL1wy0L2yHvtyaeHbnfgDOvwBHrxAJfwnaebbnrfifHhDYfgasaacH8akY=wiFfYdH8Gipec8Eeeu0xXdbba9frFj0=OqFfea0dXdd9vqai=hGuQ8kuc9pgc9s8qqaq=dirpe0xb9q8qiLsFr0=vr0=vr0dc8meaabaqaciaacaGaaeqabaWaaeGaeaaakeaaimaacqWFZestaaa@3790@) = ∫ *d****θ ****P*(**D**|ℳ
 MathType@MTEF@5@5@+=feaafiart1ev1aaatCvAUfKttLearuWrP9MDH5MBPbIqV92AaeXatLxBI9gBamrtHrhAL1wy0L2yHvtyaeHbnfgDOvwBHrxAJfwnaebbnrfifHhDYfgasaacH8akY=wiFfYdH8Gipec8Eeeu0xXdbba9frFj0=OqFfea0dXdd9vqai=hGuQ8kuc9pgc9s8qqaq=dirpe0xb9q8qiLsFr0=vr0=vr0dc8meaabaqaciaacaGaaeqabaWaaeGaeaaakeaaimaacqWFZestaaa@3790@,***θ***)*P*(***θ***|ℳ
 MathType@MTEF@5@5@+=feaafiart1ev1aaatCvAUfKttLearuWrP9MDH5MBPbIqV92AaeXatLxBI9gBamrtHrhAL1wy0L2yHvtyaeHbnfgDOvwBHrxAJfwnaebbnrfifHhDYfgasaacH8akY=wiFfYdH8Gipec8Eeeu0xXdbba9frFj0=OqFfea0dXdd9vqai=hGuQ8kuc9pgc9s8qqaq=dirpe0xb9q8qiLsFr0=vr0=vr0dc8meaabaqaciaacaGaaeqabaWaaeGaeaaakeaaimaacqWFZestaaa@3790@),     (2)

where ***θ ***is a vector of parameters of the conditional probability distributions. The marginal likelihood is an average of the likelihood *P*(**D**|ℳ
 MathType@MTEF@5@5@+=feaafiart1ev1aaatCvAUfKttLearuWrP9MDH5MBPbIqV92AaeXatLxBI9gBamrtHrhAL1wy0L2yHvtyaeHbnfgDOvwBHrxAJfwnaebbnrfifHhDYfgasaacH8akY=wiFfYdH8Gipec8Eeeu0xXdbba9frFj0=OqFfea0dXdd9vqai=hGuQ8kuc9pgc9s8qqaq=dirpe0xb9q8qiLsFr0=vr0=vr0dc8meaabaqaciaacaGaaeqabaWaaeGaeaaakeaaimaacqWFZestaaa@3790@, ***θ***) over all possible parameters assigned to the DBN with structure ℳ
 MathType@MTEF@5@5@+=feaafiart1ev1aaatCvAUfKttLearuWrP9MDH5MBPbIqV92AaeXatLxBI9gBamrtHrhAL1wy0L2yHvtyaeHbnfgDOvwBHrxAJfwnaebbnrfifHhDYfgasaacH8akY=wiFfYdH8Gipec8Eeeu0xXdbba9frFj0=OqFfea0dXdd9vqai=hGuQ8kuc9pgc9s8qqaq=dirpe0xb9q8qiLsFr0=vr0=vr0dc8meaabaqaciaacaGaaeqabaWaaeGaeaaakeaaimaacqWFZestaaa@3790@. If a certain parameter set is not highly supported by the data, Equation 2 will penalize conditional probabilities with many parameters, i.e. a node with many incoming edges or in case of discrete DBNs many states. Thus, Equation 2 matches the complexity of a model to the data size [40]. Two types of DBNs have been applied in this study: discrete and linear Gaussian DBNs. Discrete DBNs model the distribution of multinomial random variables. Before the application of discrete DBNs, the gene expression data is discretized. We either use binary quantile discretization or discretization approaches which rely on an information-preserving coalescence of discretization levels [34]. The marginal likelihood of a given model structure is computed using standard approaches [39,41]. Linear-Gaussian DBNs model the conditional distribution of Gaussian random variables assuming a linear dependence between a variable *x*_*i *_at time *t *+ 1 and its parents *pa*[*x*_*i*_] at time *t*:

xit+1=∑j=1pa[xi]bjxjt+εit;εit~N(0,σi).     (3)
 MathType@MTEF@5@5@+=feaafiart1ev1aaatCvAUfKttLearuWrP9MDH5MBPbIqV92AaeXatLxBI9gBaebbnrfifHhDYfgasaacH8akY=wiFfYdH8Gipec8Eeeu0xXdbba9frFj0=OqFfea0dXdd9vqai=hGuQ8kuc9pgc9s8qqaq=dirpe0xb9q8qiLsFr0=vr0=vr0dc8meaabaqaciaacaGaaeqabaqabeGadaaakeaafaqabeqacaaabaGaemiEaG3aa0baaSqaaiabdMgaPbqaaiabdsha0jabgUcaRiabigdaXaaakiabg2da9maaqahabaGaemOyai2aaSbaaSqaaiabdQgaQbqabaGccqWG4baEdaqhaaWcbaGaemOAaOgabaGaemiDaqhaaOGaey4kaSccciGae8xTdu2aa0baaSqaaiabdMgaPbqaaiabdsha0baaaeaacqWGQbGAcqGH9aqpcqaIXaqmaeaacqWGWbaCcqWGHbqycqGGBbWwcqWG4baEdaWgaaadbaGaemyAaKgabeaaliabc2faDbqdcqGHris5aOGaei4oaSdabaGae8xTdu2aa0baaSqaaiabdMgaPbqaaiabdsha0baakiabc6ha+jabd6eaojabcIcaOiabicdaWiabcYcaSiab=n8aZnaaBaaaleaacqWGPbqAaeqaaOGaeiykaKcaaiabc6caUiaaxMaacaWLjaWaaeWaaeaacqaIZaWmaiaawIcacaGLPaaaaaa@6288@

The R-package "Deal" is used for the calculation of the corresponding marginal likelihood [42]. The code is adopted in order to regress successive time points. For a detailed discussion on the derivation of the formulas of the marginal likelihood for multinomial and linear Gaussian conditional probability distributions see e.g. [42,43]. For a general introduction to DBNs see e.g. [40].

#### Bayesian score using knock-out data

The Bayesian score assumes that all data stems from the same underlying DBN. Because the underlying network structure is changed by gene knock-outs the Bayesian score must be adopted for the use of knock-out data [44,45]. Due to its decomposability, the Bayesian score factorizes into terms for each variable. Therefore, the total network score can be computed by considering each variable *x*_*i *_and its parents *pa*[*x*_*i*_] together with the appropriate subset of data Dxi,pa[xi]
 MathType@MTEF@5@5@+=feaafiart1ev1aaatCvAUfKttLearuWrP9MDH5MBPbIqV92AaeXatLxBI9gBaebbnrfifHhDYfgasaacH8akY=wiFfYdH8Gipec8Eeeu0xXdbba9frFj0=OqFfea0dXdd9vqai=hGuQ8kuc9pgc9s8qqaq=dirpe0xb9q8qiLsFr0=vr0=vr0dc8meaabaqaciaacaGaaeqabaqabeGadaaakeaaieqacqWFebardaWgaaWcbaGaemiEaG3aaSbaaWqaaiabdMgaPbqabaWccqGGSaalcqWGWbaCcqWGHbqycqGGBbWwcqWG4baEdaWgaaadbaGaemyAaKgabeaaliabc2faDbqabaaaaa@3A1B@ separately. Manipulating the state of variable *x*_*i *_to a certain value *k *changes its conditional probability distribution:

P(xi|pa[xi])→P(xi)={1if xi=k0otherwise.     (4)
 MathType@MTEF@5@5@+=feaafiart1ev1aaatCvAUfKttLearuWrP9MDH5MBPbIqV92AaeXatLxBI9gBaebbnrfifHhDYfgasaacH8akY=wiFfYdH8Gipec8Eeeu0xXdbba9frFj0=OqFfea0dXdd9vqai=hGuQ8kuc9pgc9s8qqaq=dirpe0xb9q8qiLsFr0=vr0=vr0dc8meaabaqaciaacaGaaeqabaqabeGadaaakeaacqWGqbaucqGGOaakcqWG4baEdaWgaaWcbaGaemyAaKgabeaakiabcYha8jabdchaWjabdggaHjabcUfaBjabdIha4naaBaaaleaacqWGPbqAaeqaaOGaeiyxa0LaeiykaKIaeyOKH4QaemiuaaLaeiikaGIaemiEaG3aaSbaaSqaaiabdMgaPbqabaGccqGGPaqkcqGH9aqpdaGabeqaauaabaqaciaaaeaacqaIXaqmaeaacqqGPbqAcqqGMbGzcqqGGaaicqWG4baEdaWgaaWcbaGaemyAaKgabeaakiabg2da9iabdUgaRbqaaiabicdaWaqaaiabb+gaVjabbsha0jabbIgaOjabbwgaLjabbkhaYjabbEha3jabbMgaPjabbohaZjabbwgaLjabc6caUaaaaiaawUhaaiaaxMaacaWLjaWaaeWaaeaacqaI0aanaiaawIcacaGLPaaaaaa@622B@

Hence, manipulating variable *x*_*i *_makes it independent of its parents. This implies that the data subset Dxi,pa[xi]
 MathType@MTEF@5@5@+=feaafiart1ev1aaatCvAUfKttLearuWrP9MDH5MBPbIqV92AaeXatLxBI9gBaebbnrfifHhDYfgasaacH8akY=wiFfYdH8Gipec8Eeeu0xXdbba9frFj0=OqFfea0dXdd9vqai=hGuQ8kuc9pgc9s8qqaq=dirpe0xb9q8qiLsFr0=vr0=vr0dc8meaabaqaciaacaGaaeqabaqabeGadaaakeaaieqacqWFebardaWgaaWcbaGaemiEaG3aaSbaaWqaaiabdMgaPbqabaWccqGGSaalcqWGWbaCcqWGHbqycqGGBbWwcqWG4baEdaWgaaadbaGaemyAaKgabeaaliabc2faDbqabaaaaa@3A1B@ used to score the conditional probability distribution *P*(*x*_*i*_|*pa*[*x*_*i*_]) must not include data of *x*_*i *_and its parents derived under manipulation of *x*_*i*_.

#### Exhaustive evaluation of all possible TF combinations

Searching for an optimal set of regulators of a given gene is often done using local optimization techniques [7]. However, with the restrictions of TFs as possible regulators and a maximum number of TFs per gene it is feasible to perform an exhaustive computation of the Bayesian scores for all possible combinations of TFs and target genes. Given *M *TFs regulating *N *genes, N∑k=0Mmax(Mk)
 MathType@MTEF@5@5@+=feaafiart1ev1aaatCvAUfKttLearuWrP9MDH5MBPbIqV92AaeXatLxBI9gBaebbnrfifHhDYfgasaacH8akY=wiFfYdH8Gipec8Eeeu0xXdbba9frFj0=OqFfea0dXdd9vqai=hGuQ8kuc9pgc9s8qqaq=dirpe0xb9q8qiLsFr0=vr0=vr0dc8meaabaqaciaacaGaaeqabaqabeGadaaakeaacqWGobGtdaaeWaqaamaabmaabaqbaeqabiqaaaqaaiabd2eanbqaaiabdUgaRbaaaiaawIcacaGLPaaaaSqaaiabdUgaRjabg2da9iabicdaWaqaaiabd2eannaaBaaameaaieGacqWFTbqBcqWFHbqycqWF4baEaeqaaaqdcqGHris5aaaa@3CB4@ TF-target gene combinations are evaluated. Here, *M*_*max *_is a prior restriction on the maximum number of TFs per target gene; in this study *M*_*max *_= 4. If *M *≪ *N*, the total number of combinations is still feasible to compute even for a large number of genes. In case of real biological data, prior knowledge derived from gene ontologies or ChIP analysis can be used in order to restrict the set of possible regulators.

### Markov chain Monte Carlo simulations

In order to update prior biological knowledge about the network structure by new data **D**, the posterior probability of network is computed using Bayes theorem:

P(ℳ|D)=1ZP(D|ℳ)P(ℳ).     (5)
 MathType@MTEF@5@5@+=feaafiart1ev1aaatCvAUfKttLearuWrP9MDH5MBPbIqV92AaeXatLxBI9gBaebbnrfifHhDYfgasaacH8akY=wiFfYdH8Gipec8Eeeu0xXdbba9frFj0=OqFfea0dXdd9vqai=hGuQ8kuc9pgc9s8qqaq=dirpe0xb9q8qiLsFr0=vr0=vr0dc8meaabaqaciaacaGaaeqabaqabeGadaaakeaacqWGqbaucqGGOaakt0uy0HwzTfgDPnwy1egaryqtHrhAL1wy0L2yHvdaiqaacqWFZestcqGG8baFieqacqGFebarcqGGPaqkcqGH9aqpdaWcaaqaaiabigdaXaqaaiabdQfaAbaacqWGqbaucqGGOaakcqGFebarcqGG8baFcqWFZestcqGGPaqkcqWGqbaucqGGOaakcqWFZestcqGGPaqkcqGGUaGlcaWLjaGaaCzcamaabmaabaGaeGynaudacaGLOaGaayzkaaaaaa@4F7A@

*P*(**D**|ℳ
 MathType@MTEF@5@5@+=feaafiart1ev1aaatCvAUfKttLearuWrP9MDH5MBPbIqV92AaeXatLxBI9gBamrtHrhAL1wy0L2yHvtyaeHbnfgDOvwBHrxAJfwnaebbnrfifHhDYfgasaacH8akY=wiFfYdH8Gipec8Eeeu0xXdbba9frFj0=OqFfea0dXdd9vqai=hGuQ8kuc9pgc9s8qqaq=dirpe0xb9q8qiLsFr0=vr0=vr0dc8meaabaqaciaacaGaaeqabaWaaeGaeaaakeaaimaacqWFZestaaa@3790@) is the marginal likelihood and *P*(ℳ
 MathType@MTEF@5@5@+=feaafiart1ev1aaatCvAUfKttLearuWrP9MDH5MBPbIqV92AaeXatLxBI9gBamrtHrhAL1wy0L2yHvtyaeHbnfgDOvwBHrxAJfwnaebbnrfifHhDYfgasaacH8akY=wiFfYdH8Gipec8Eeeu0xXdbba9frFj0=OqFfea0dXdd9vqai=hGuQ8kuc9pgc9s8qqaq=dirpe0xb9q8qiLsFr0=vr0=vr0dc8meaabaqaciaacaGaaeqabaWaaeGaeaaakeaaimaacqWFZestaaa@3790@) is a structural prior which can be used to include prior knowledge on gene-gene interactions. It can be derived from additional data sources as e.g. promotor analysis or ChIP experiments. The normalization constant *Z *is given by ∑ℳP(D|ℳ)P(ℳ)
 MathType@MTEF@5@5@+=feaafiart1ev1aaatCvAUfKttLearuWrP9MDH5MBPbIqV92AaeXatLxBI9gBaebbnrfifHhDYfgasaacH8akY=wiFfYdH8Gipec8Eeeu0xXdbba9frFj0=OqFfea0dXdd9vqai=hGuQ8kuc9pgc9s8qqaq=dirpe0xb9q8qiLsFr0=vr0=vr0dc8meaabaqaciaacaGaaeqabaqabeGadaaakeaadaaeqaqaaiabdcfaqjabcIcaOGqabiab=reaejabcYha8nrtHrhAL1wy0L2yHvtyaeHbnfgDOvwBHrxAJfwnaGabaiab+ntinjabcMcaPiabdcfaqjabcIcaOiab+ntinjabcMcaPaWcbaGae43mH0eabeqdcqGHris5aaaa@4408@. For large models, i.e. more than 4 variables, it is not feasible to compute *Z *by exhaustive methods as for DBNs with *N *variables the corresponding model space includes 2N2
 MathType@MTEF@5@5@+=feaafiart1ev1aaatCvAUfKttLearuWrP9MDH5MBPbIqV92AaeXatLxBI9gBaebbnrfifHhDYfgasaacH8akY=wiFfYdH8Gipec8Eeeu0xXdbba9frFj0=OqFfea0dXdd9vqai=hGuQ8kuc9pgc9s8qqaq=dirpe0xb9q8qiLsFr0=vr0=vr0dc8meaabaqaciaacaGaaeqabaqabeGadaaakeaacqaIYaGmdaahaaWcbeqaaiabd6eaonaaCaaameqabaGaeGOmaidaaaaaaaa@3010@ models. Markov chain Monte Carlo (MCMC) simulations are used to approximate posterior probabilities of model features, i.e. gene-gene interactions, by frequencies of samples taken from a random walk in model space [21,46]. The Markov chain is started with the best model (see above) making an equilibration of the chain unnecessary. In each step a new TF combination for a given gene is chosen randomly (termed new model). The Metropolis-Hastings acceptance criterion is used to determine the acceptance probability *A *of the new model:

A(ℳold→ℳnew)={cif c<11otherwise     (6)
 MathType@MTEF@5@5@+=feaafiart1ev1aaatCvAUfKttLearuWrP9MDH5MBPbIqV92AaeXatLxBI9gBaebbnrfifHhDYfgasaacH8akY=wiFfYdH8Gipec8Eeeu0xXdbba9frFj0=OqFfea0dXdd9vqai=hGuQ8kuc9pgc9s8qqaq=dirpe0xb9q8qiLsFr0=vr0=vr0dc8meaabaqaciaacaGaaeqabaqabeGadaaakeaacqWGbbqqcqGGOaakt0uy0HwzTfgDPnwy1egaryqtHrhAL1wy0L2yHvdaiqaacqWFZestdaWgaaWcbaGaem4Ba8MaemiBaWMaemizaqgabeaakiabgkziUkab=ntinnaaBaaaleaacqWGUbGBcqWGLbqzcqWG3bWDaeqaaOGaeiykaKIaeyypa0ZaaiqabeaafaqaaeGacaaabaGaem4yamgabaGaeeyAaKMaeeOzayMaeeiiaaIaem4yamMaeyipaWJaeGymaedabaGaeGymaedabaGaee4Ba8MaeeiDaqNaeeiAaGMaeeyzauMaeeOCaiNaee4DaCNaeeyAaKMaee4CamNaeeyzaugaaaGaay5EaaGaaCzcaiaaxMaadaqadaqaaiabiAda2aGaayjkaiaawMcaaaaa@6172@

where

c=P(D|ℳnew)P(D|ℳold)P(ℳnew)P(ℳold).     (7)
 MathType@MTEF@5@5@+=feaafiart1ev1aaatCvAUfKttLearuWrP9MDH5MBPbIqV92AaeXatLxBI9gBaebbnrfifHhDYfgasaacH8akY=wiFfYdH8Gipec8Eeeu0xXdbba9frFj0=OqFfea0dXdd9vqai=hGuQ8kuc9pgc9s8qqaq=dirpe0xb9q8qiLsFr0=vr0=vr0dc8meaabaqaciaacaGaaeqabaqabeGadaaakeaacqWGJbWycqGH9aqpdaWcaaqaaiabdcfaqjabcIcaOGqabiab=reaejabcYha8nrtHrhAL1wy0L2yHvtyaeHbnfgDOvwBHrxAJfwnaGabaiab+ntinnaaBaaaleaacqWGUbGBcqWGLbqzcqWG3bWDaeqaaOGaeiykaKcabaGaemiuaaLaeiikaGIae8hraqKaeiiFaWNae43mH00aaSbaaSqaaiabd+gaVjabdYgaSjabdsgaKbqabaGccqGGPaqkaaWaaSaaaeaacqWGqbaucqGGOaakcqGFZestdaWgaaWcbaGaemOBa4MaemyzauMaem4DaChabeaakiabcMcaPaqaaiabdcfaqjabcIcaOiab+ntinnaaBaaaleaacqWGVbWBcqWGSbaBcqWGKbazaeqaaOGaeiykaKcaaiabc6caUiaaxMaacaWLjaWaaeWaaeaacqaI3aWnaiaawIcacaGLPaaaaaa@6417@

Since the proposal probabilities for an old vs. a new model are equal they are omitted in Equation 7. For a given network of *N *genes the Markov chain is sampled in *N*^2 ^steps (≡1 MCMC cycle) in order to achieve an independence of successive samples. The convergence of the Markov chain is monitored during run-time (see below). After convergence, the final posterior probabilities of the model features are determined by averaging across samples,

P(k)≈1L∑i=1Lfk(ℳi),     (8)
 MathType@MTEF@5@5@+=feaafiart1ev1aaatCvAUfKttLearuWrP9MDH5MBPbIqV92AaeXatLxBI9gBaebbnrfifHhDYfgasaacH8akY=wiFfYdH8Gipec8Eeeu0xXdbba9frFj0=OqFfea0dXdd9vqai=hGuQ8kuc9pgc9s8qqaq=dirpe0xb9q8qiLsFr0=vr0=vr0dc8meaabaqaciaacaGaaeqabaqabeGadaaakeaacqWGqbaucqGGOaakcqWGRbWAcqGGPaqkcqGHijYUdaWcaaqaaiabigdaXaqaaiabdYeambaadaaeWbqaaiabdAgaMnaaBaaaleaacqWGRbWAaeqaaOGaeiikaGYenfgDOvwBHrxAJfwnHbqeg0uy0HwzTfgDPnwy1aaceaGae83mH00aaSbaaSqaaiabdMgaPbqabaGccqGGPaqkaSqaaiabdMgaPjabg2da9iabigdaXaqaaiabdYeambqdcqGHris5aOGaeiilaWIaaCzcaiaaxMaadaqadaqaaiabiIda4aGaayjkaiaawMcaaaaa@5131@

where *L *is the sample number and *f*_*k*_(·) is the indicator function for feature *k*.

#### Monitoring MCMC convergence

As an indicator for the convergence of the Markov chain the variance of edge probabilities is monitored by block-averaging during run-time. Running the MCMC simulation from *t*_0 _up to *t*_*n *_for a decade of *n *MCMC cycles, this decade is divided into *b *blocks of length nb
 MathType@MTEF@5@5@+=feaafiart1ev1aaatCvAUfKttLearuWrP9MDH5MBPbIqV92AaeXatLxBI9gBaebbnrfifHhDYfgasaacH8akY=wiFfYdH8Gipec8Eeeu0xXdbba9frFj0=OqFfea0dXdd9vqai=hGuQ8kuc9pgc9s8qqaq=dirpe0xb9q8qiLsFr0=vr0=vr0dc8meaabaqaciaacaGaaeqabaqabeGadaaakeaadaWcaaqaaiabd6gaUbqaaiabdkgaIbaaaaa@2F6E@. For each block *i *the block mean of each edge *X*^*j *^is calculated from its nb
 MathType@MTEF@5@5@+=feaafiart1ev1aaatCvAUfKttLearuWrP9MDH5MBPbIqV92AaeXatLxBI9gBaebbnrfifHhDYfgasaacH8akY=wiFfYdH8Gipec8Eeeu0xXdbba9frFj0=OqFfea0dXdd9vqai=hGuQ8kuc9pgc9s8qqaq=dirpe0xb9q8qiLsFr0=vr0=vr0dc8meaabaqaciaacaGaaeqabaqabeGadaaakeaadaWcaaqaaiabd6gaUbqaaiabdkgaIbaaaaa@2F6E@ block samples:

Xij=bn∑t=[i−1]tnb[itnb]−1Xj(t).     (9)
 MathType@MTEF@5@5@+=feaafiart1ev1aaatCvAUfKttLearuWrP9MDH5MBPbIqV92AaeXatLxBI9gBaebbnrfifHhDYfgasaacH8akY=wiFfYdH8Gipec8Eeeu0xXdbba9frFj0=OqFfea0dXdd9vqai=hGuQ8kuc9pgc9s8qqaq=dirpe0xb9q8qiLsFr0=vr0=vr0dc8meaabaqaciaacaGaaeqabaqabeGadaaakeaacqWGybawdaqhaaWcbaGaemyAaKgabaGaemOAaOgaaOGaeyypa0ZaaSaaaeaacqWGIbGyaeaacqWGUbGBaaWaaabCaeaacqWGybawdaahaaWcbeqaaiabdQgaQbaakiabcIcaOiabdsha0jabcMcaPaWcbaGaemiDaqNaeyypa0Jaei4waSLaemyAaKMaeyOeI0IaeGymaeJaeiyxa01aaSaaaeaacqWG0baDdaWgaaadbaGaemOBa4gabeaaaSqaaiabdkgaIbaaaeaacqGGBbWwcqWGPbqAdaWcaaqaaiabdsha0naaBaaameaacqWGUbGBaeqaaaWcbaGaemOyaigaaiabc2faDjabgkHiTiabigdaXaqdcqGHris5aOGaeiOla4IaaCzcaiaaxMaadaqadaqaaiabiMda5aGaayjkaiaawMcaaaaa@5846@

The overall decade mean and variance of each edge *X*^*j *^is then calculated from its *b *block means:

X¯j=1b∑i=1bXij     (10)
 MathType@MTEF@5@5@+=feaafiart1ev1aaatCvAUfKttLearuWrP9MDH5MBPbIqV92AaeXatLxBI9gBaebbnrfifHhDYfgasaacH8akY=wiFfYdH8Gipec8Eeeu0xXdbba9frFj0=OqFfea0dXdd9vqai=hGuQ8kuc9pgc9s8qqaq=dirpe0xb9q8qiLsFr0=vr0=vr0dc8meaabaqaciaacaGaaeqabaqabeGadaaakeaacuWGybawgaqeamaaCaaaleqabaGaemOAaOgaaOGaeyypa0ZaaSaaaeaacqaIXaqmaeaacqWGIbGyaaWaaabCaeaacqWGybawdaqhaaWcbaGaemyAaKgabaGaemOAaOgaaaqaaiabdMgaPjabg2da9iabigdaXaqaaiabdkgaIbqdcqGHris5aOGaaCzcaiaaxMaadaqadaqaaiabigdaXiabicdaWaGaayjkaiaawMcaaaaa@428C@

Var(Xj)=1b∑i=1b(Xij−X¯j)2.     (11)
 MathType@MTEF@5@5@+=feaafiart1ev1aaatCvAUfKttLearuWrP9MDH5MBPbIqV92AaeXatLxBI9gBaebbnrfifHhDYfgasaacH8akY=wiFfYdH8Gipec8Eeeu0xXdbba9frFj0=OqFfea0dXdd9vqai=hGuQ8kuc9pgc9s8qqaq=dirpe0xb9q8qiLsFr0=vr0=vr0dc8meaabaqaciaacaGaaeqabaqabeGadaaakeaacqWGwbGvcqWGHbqycqWGYbGCcqGGOaakcqWGybawdaahaaWcbeqaaiabdQgaQbaakiabcMcaPiabg2da9maalaaabaGaeGymaedabaGaemOyaigaamaaqahabaGaeiikaGIaemiwaG1aa0baaSqaaiabdMgaPbqaaiabdQgaQbaakiabgkHiTiqbdIfayzaaraWaaWbaaSqabeaacqWGQbGAaaGccqGGPaqkdaahaaWcbeqaaiabikdaYaaaaeaacqWGPbqAcqGH9aqpcqaIXaqmaeaacqWGIbGya0GaeyyeIuoakiabc6caUiaaxMaacaWLjaWaaeWaaeaacqaIXaqmcqaIXaqmaiaawIcacaGLPaaaaaa@4FA6@

The procedure is initialized with a decade size of *n *= *b *and in successive decades the size is updated as *n*_*new *_= *b *× *n*_*old*_. The mean of the last decade can be used as the mean of the first block in the new decade. The MCMC simulations in the present study run for about 10^6 ^MCMC cycles to achieve an variance of all edge probabilities ≤ 10^-4^.

#### Reducing model space

Data size as well as constraints on the maximal number of parents per node can lead the MCMC simulation to stay in a restricted area of model space for a long time. As a negative side effect some edges are never altered giving rise to misleading posterior probabilities of either 0 or 1 with a variance of 0. To circumvent these difficulties two strategies are used to restrict the model space and thereby improving MCMC convergence rates:

1. In case of data coming from the set of synthetic networks only TFs can be regulators of a gene in the network.

2. Due to its decomposability the Bayesian score of DBNs can be pre-calculated for each possible TF target gene combination before the MCMC simulation in model space is started. For each gene *x*_*i *_the Bayesian score Si∗
 MathType@MTEF@5@5@+=feaafiart1ev1aaatCvAUfKttLearuWrP9MDH5MBPbIqV92AaeXatLxBI9gBaebbnrfifHhDYfgasaacH8akY=wiFfYdH8Gipec8Eeeu0xXdbba9frFj0=OqFfea0dXdd9vqai=hGuQ8kuc9pgc9s8qqaq=dirpe0xb9q8qiLsFr0=vr0=vr0dc8meaabaqaciaacaGaaeqabaqabeGadaaakeaacqWGtbWudaqhaaWcbaGaemyAaKgabaGaey4fIOcaaaaa@3052@ of its best parent combination *pa*[*x*_*i*_]* is determined. The model space **M**_*i *_for gene *x*_*i *_is reduced by excluding all parent combination for gene *x*_*i *_with a score below a given threshold, i.e.,

MiRed={pa[xi]∈Mi|S(xi|pa[xi])≥αSi∗}     (12)
 MathType@MTEF@5@5@+=feaafiart1ev1aaatCvAUfKttLearuWrP9MDH5MBPbIqV92AaeXatLxBI9gBaebbnrfifHhDYfgasaacH8akY=wiFfYdH8Gipec8Eeeu0xXdbba9frFj0=OqFfea0dXdd9vqai=hGuQ8kuc9pgc9s8qqaq=dirpe0xb9q8qiLsFr0=vr0=vr0dc8meaabaqaciaacaGaaeqabaqabeGadaaakeaaieqacqWFnbqtdaqhaaWcbaGaemyAaKgabaacbiGae4NuaiLae4xzauMaemizaqgaaOGaeyypa0Jaei4EaSNaemiCaaNaemyyaeMaei4waSLaemiEaG3aaSbaaSqaaiabdMgaPbqabaGccqGGDbqxcqGHiiIZcqWFnbqtdaWgaaWcbaGaemyAaKgabeaakiabcYha8jabdofatjabcIcaOiabdIha4naaBaaaleaacqWGPbqAaeqaaOGaeiiFaWNaemiCaaNaemyyaeMaei4waSLaemiEaG3aaSbaaSqaaiabdMgaPbqabaGccqGGDbqxcqGGPaqkcqGHLjYSiiGacqqFXoqycqWGtbWudaqhaaWcbaGaemyAaKgabaGaey4fIOcaaOGaeiyFa0NaaCzcaiaaxMaadaqadaqaaiabigdaXiabikdaYaGaayjkaiaawMcaaaaa@60A3@

where *α *∈ [0,1]. In this way it is possible to focus only on models with high posterior probabilities. The convergence of the MCMC simulation can be accelerated by a factor of 10 while at the same time computing accurate posterior probabilities (data not shown).

#### Variable selection based on F-statistics

Variable selection is a common method in regression analysis used to reduce a potentially large set of regressors. In order to identify a potential set of TFs for each gene step-wise forward selection and backward elimination of variables (i.e. TFs) is used. The selection/elimination criterion is based on a partial F-test. In each step the variable with the most significant partial correlation coefficient is selected while the variable with the smallest and non-significant partial F-statistics is removed. The final set of TFs is filtered for variables with a p-value of the partial correlation coefficient *p *<= 0.01. Variable selection is performed with the functions 1 m and step of the statistical computing environment R [47].

### Random networks and data generation

Each network is represented by a random connectivity matrix of size 10 TF × 30 genes. The connectivity matrix is constructed by sampling the number of TFs for each of the 30 genes from uniform distribution: 1 ≤ #*TFs *≤ *M*_*max*_. The TFs are randomly connected each having either activatory or inhibitory effect on the regulation of the target gene. Two different types of network dynamics are simulated.

• In the ODE setting, the expression dynamics of a gene is modelled by a constant activation, a first-order degradation and a saturating Hill function which combines activation and repression of the gene by its regulators. Time delays, due to the transcription and translation of a gene, are not taken into account. All TFs are assumed to act as homo-dimers. The regulatory strength of TF *j *onto target gene *i *is given by a randomly chosen coefficient -1 ≤ *α*_*ij *_≤ 1. The corresponding system of coupled non-linear ODEs is given by,

x˙i=bi+∑j∈Aiαijxi2K+∑j∈Aiαijxj2+∑k∈Riαikxk2−λixi     (13)
 MathType@MTEF@5@5@+=feaafiart1ev1aaatCvAUfKttLearuWrP9MDH5MBPbIqV92AaeXatLxBI9gBaebbnrfifHhDYfgasaacH8akY=wiFfYdH8Gipec8Eeeu0xXdbba9frFj0=OqFfea0dXdd9vqai=hGuQ8kuc9pgc9s8qqaq=dirpe0xb9q8qiLsFr0=vr0=vr0dc8meaabaqaciaacaGaaeqabaqabeGadaaakeaacuWG4baEgaGaamaaBaaaleaacqWGPbqAaeqaaOGaeyypa0JaemOyai2aaSbaaSqaaiabdMgaPbqabaGccqGHRaWkdaWcaaqaamaaqababaacciGae8xSde2aaSbaaSqaaiabdMgaPjabdQgaQbqabaGccqWG4baEdaqhaaWcbaGaemyAaKgabaGaeGOmaidaaaqaaiabdQgaQjabgIGiolabdgeabnaaBaaameaacqWGPbqAaeqaaaWcbeqdcqGHris5aaGcbaGaem4saSKaey4kaSYaaabeaeaacqWFXoqydaWgaaWcbaGaemyAaKMaemOAaOgabeaakiabdIha4naaDaaaleaacqWGQbGAaeaacqaIYaGmaaaabaGaemOAaOMaeyicI4Saemyqae0aaSbaaWqaaiabdMgaPbqabaaaleqaniabggHiLdGccqGHRaWkdaaeqaqaaiab=f7aHnaaBaaaleaacqWGPbqAcqWGRbWAaeqaaOGaemiEaG3aa0baaSqaaiabdUgaRbqaaiabikdaYaaaaeaacqWGRbWAcqGHiiIZcqWGsbGudaWgaaadbaGaemyAaKgabeaaaSqab0GaeyyeIuoaaaGccqGHsislcqWF7oaBdaWgaaWcbaGaemyAaKgabeaakiabdIha4naaBaaaleaacqWGPbqAaeqaaOGaaCzcaiaaxMaadaqadaqaaiabigdaXiabiodaZaGaayjkaiaawMcaaaaa@732C@

where *x*_*i *_is the expression level of gene *i *and *A*_*i *_and *R*_*i *_are the sets of activating and inhibiting TFs of gene *i *respectively as defined by the connectivity matrix. Each gene has a constant activation term *b*_*i *_= 5 and is self-regulated by a degradation term with strength *λ*_*i *_= 0.5. The value of *K *is equal to the sum of activator and inhibitor concentrations for which a half-maximal transcription rate is achieved; it is set to *K *= 1 for all genes. The gene expression model is coupled to an observation model which accounts for additive-multiplicative Gaussian noise [30]:

*y*_*i *_= *ε*_*a *_+ *x*_*i*_(1 + *ε*_*m*_); *ε*_*a*/*m *_~ *N*(0, *ρ*).     (14)

For simplicity the standard deviation *ρ *of the additive noise term *σ*_*a *_and of the multiplicative noise term *σ*_*m *_is chosen to be equal. *ρ *is ranging between 0.02 ≤ *ρ *≤ 0.2. The steady state of the system of ODEs is determined numerically. After perturbation from steady state, the system is sampled during relaxation back to steady state. A KO of a gene is simulated by setting its expression level to zero during the integration.

• In a Boolean network the sign of the coefficients of the connectivity matrix identify a TF either as an activator (changing the state of a target according to [0,1] → 1) or as an inhibitor ([0,1] → 0). The action of activators and inhibitors is combined by a logical OR gate. Time series are generated starting with a random initial state of the networks. The effect of noise is simulated by randomly flipping the outcome of the logical OR gate with a probability according to the noise strength.

### Calculation of error rates

Three error rates of the network reconstruction are calculated. The false-negative (FN) rate, false-positive (FP) rate and realized false discovery rate (FDR) are given by:

FNR=#FN#TP+#FN     (15)
 MathType@MTEF@5@5@+=feaafiart1ev1aaatCvAUfKttLearuWrP9MDH5MBPbIqV92AaeXatLxBI9gBaebbnrfifHhDYfgasaacH8akY=wiFfYdH8Gipec8Eeeu0xXdbba9frFj0=OqFfea0dXdd9vqai=hGuQ8kuc9pgc9s8qqaq=dirpe0xb9q8qiLsFr0=vr0=vr0dc8meaabaqaciaacaGaaeqabaqabeGadaaakeaacqWGgbGrcqWGobGtcqWGsbGucqGH9aqpdaWcaaqaaiabcocaJiabdAeagjabd6eaobqaaiabcocaJiabdsfaujabdcfaqjabgUcaRiabcocaJiabdAeagjabd6eaobaacaWLjaGaaCzcamaabmaabaGaeGymaeJaeGynaudacaGLOaGaayzkaaaaaa@3FF8@

FPR=#FP#FP+#TN     (16)
 MathType@MTEF@5@5@+=feaafiart1ev1aaatCvAUfKttLearuWrP9MDH5MBPbIqV92AaeXatLxBI9gBaebbnrfifHhDYfgasaacH8akY=wiFfYdH8Gipec8Eeeu0xXdbba9frFj0=OqFfea0dXdd9vqai=hGuQ8kuc9pgc9s8qqaq=dirpe0xb9q8qiLsFr0=vr0=vr0dc8meaabaqaciaacaGaaeqabaqabeGadaaakeaacqWGgbGrcqWGqbaucqWGsbGucqGH9aqpdaWcaaqaaiabcocaJiabdAeagjabdcfaqbqaaiabcocaJiabdAeagjabdcfaqjabgUcaRiabcocaJiabdsfaujabd6eaobaacaWLjaGaaCzcamaabmaabaGaeGymaeJaeGOnaydacaGLOaGaayzkaaaaaa@4002@

FDR=#FP#FP+#TP.     (17)
 MathType@MTEF@5@5@+=feaafiart1ev1aaatCvAUfKttLearuWrP9MDH5MBPbIqV92AaeXatLxBI9gBaebbnrfifHhDYfgasaacH8akY=wiFfYdH8Gipec8Eeeu0xXdbba9frFj0=OqFfea0dXdd9vqai=hGuQ8kuc9pgc9s8qqaq=dirpe0xb9q8qiLsFr0=vr0=vr0dc8meaabaqaciaacaGaaeqabaqabeGadaaakeaacqWGgbGrcqWGebarcqWGsbGucqGH9aqpdaWcaaqaaiabcocaJiabdAeagjabdcfaqbqaaiabcocaJiabdAeagjabdcfaqjabgUcaRiabcocaJiabdsfaujabdcfaqbaacqGGUaGlcaWLjaGaaCzcamaabmaabaGaeGymaeJaeG4naCdacaGLOaGaayzkaaaaaa@40D4@

FN, FP, true-negatives (TN) and true-positives (TP) are based on a edge-wise comparison of the best predicted network with the true network.

### Performance on a network with hidden variables

The gene-regulatory network proposed by [31] was used to examine the effect of hidden variables on the performance of network reconstruction. The complete connectivity matrix of the dynamical model proposed by [31] is defined by all non-zero entries of the corresponding Jacobian matrix:

A(i,j)={1if ∂x˙j∂xi≠00otherwise.     (18)
 MathType@MTEF@5@5@+=feaafiart1ev1aaatCvAUfKttLearuWrP9MDH5MBPbIqV92AaeXatLxBI9gBaebbnrfifHhDYfgasaacH8akY=wiFfYdH8Gipec8Eeeu0xXdbba9frFj0=OqFfea0dXdd9vqai=hGuQ8kuc9pgc9s8qqaq=dirpe0xb9q8qiLsFr0=vr0=vr0dc8meaabaqaciaacaGaaeqabaqabeGadaaakeaacqWGbbqqcqGGOaakcqWGPbqAcqGGSaalcqWGQbGAcqGGPaqkcqGH9aqpdaGabeqaauaabaqaciaaaeaacqaIXaqmaeaacqqGPbqAcqqGMbGzcqqGGaaidaWcaaqaaiabgkGi2kqbdIha4zaacaWaaSbaaSqaaiabdQgaQbqabaaakeaacqGHciITcqWG4baEdaWgaaWcbaGaemyAaKgabeaaaaGccqGHGjsUcqaIWaamaeaacqaIWaamaeaacqqGVbWBcqqG0baDcqqGObaAcqqGLbqzcqqGYbGCcqqG3bWDcqqGPbqAcqqGZbWCcqqGLbqzcqGGUaGlaaaacaGL7baacaWLjaGaaCzcamaabmaabaGaeGymaeJaeGioaGdacaGLOaGaayzkaaaaaa@5844@

From this complete connectivity matrix we construct a smaller matrix connecting only an observed subset of variables, i.e. mRNA states in the present case. For each hidden variable *X *∉ {mRNAs} the row and column corresponding to *X *is removed from *A *and all parent-variables of *X *are connected with all children of *X*. In this way all indirect regulations are preserved. The reduced connectivity matrix *A*^*obs *^for a given set of observed variables is unique and the order of removing nodes from the full network is irrelevant.

## Authors' contributions

FG performed the simulations and drafted the manuscript. CF supervised the work and drafted the manuscript. JT initiated the work and help to draft the manuscript. All authors read and approved the final manuscript.
